# Acquisition and transfer of antibiotic resistance genes in association with conjugative plasmid or class 1 integrons of *Acinetobacter baumannii*

**DOI:** 10.1371/journal.pone.0208468

**Published:** 2018-12-06

**Authors:** Udomluk Leungtongkam, Rapee Thummeepak, Kannipa Tasanapak, Sutthirat Sitthisak

**Affiliations:** 1 Department of Microbiology and Parasitology, Faculty of Medical Science, Naresuan University, Phitsanulok, Thailand; 2 Centre of Excellence in Medical Biotechnology, Naresuan University, Phitsanulok, Thailand; Northwestern University Feinberg School of Medicine, UNITED STATES

## Abstract

Conjugation is a type of horizontal gene transfer (HGT) that serves as the primary mechanism responsible for accelerating the spread of antibiotic resistance genes in Gram-negative bacteria. The present study aimed to elucidate the mechanisms underlying the conjugation-mediated gene transfer from the extensively drug-resistant *Acinetobacter baumannii* (XDR-AB) and New Delhi Metallo-beta-lactamase-1-producing *Acinetobacter baumannii* (NDM-AB) to environmental isolates of *Acinetobacter* spp. Conjugation experiments demonstrated that resistance to ticarcillin and kanamycin could be transferred from four donors to two sodium azide-resistant *A*. *baumannii* strains, namely, NU013R and NU015R. No transconjugants were detected on Mueller-Hinton Agar (MHA) plates containing tetracycline. Plasmids obtained from donors as well as successful transconjugants were characterized by PCR-based replicon typing and S1-nuclease pulsed-field gel electrophoresis (S1-PFGE). Detection of antibiotic resistance genes and integrase genes (*int*) was performed using PCR. Results revealed that the donor AB364 strain can transfer the *bla*_OXA-23_ and *bla*_PER-1_ genes to both recipients in association with *int1*. A 240-kb plasmid was successfully transferred from the donor AB364 to recipients. In addition, the *aphA6* and *bla*_PER-1_ genes were co-transferred with the *int1* gene from the donor strains AB352 and AB405. The transfer of a 220-kb plasmid from the donors to recipient was detected. The GR6 plasmid containing the kanamycin resistance gene (*aphA6*) was successfully transferred from the donor strain AB140 to both recipient strains. However, the *bla*_NDM-1_ and *tet*(B) genes were not detected in all transconjugants. Our study is the first to demonstrate successful *in vitro* conjugation, which indicated that XDR-AB contained combination mechanisms of the co-transfer of antimicrobial resistance elements with integron cassettes or with the plasmid group GR6. Thus, conjugation could be responsible for the emergence of new types of antibiotic-resistant strains.

## Introduction

The genus *Acinetobacter* comprises important human pathogens that cause nosocomial infections in immunocompromised hosts. The emergence of extensively drug-resistant *A*. *baumannii* (XDR-AB) and New Delhi Metallo-beta-lactamase-1-producing *A*. *baumannii* (NDM-AB) is a major and immediate threat to public health worldwide. The production of β-lactamase enzymes, including class A-D enzymes, is the primary mechanism underlying *A*. *baumannii* resistance [1–3]. The primary mechanism underlying aminoglycoside resistance in *A*. *baumannii* is enzymatic inactivation by acetyltransferases (AAC) [4]. Additionally, the presence of acquired efflux pumps has been reported in *Acinetobacter* spp. Several Tet efflux pumps, including *tet***(**A**)** and *tet*(B), which confer tetracycline resistance, have been acquired by clinical isolates of *A*. *baumannii* [5].

Mobile genetic elements (MGEs), such as plasmids, integrons, and transposons, have been identified as sources of multidrug resistance in *A*. *baumannii* [6]. Integrons are mobile genetic elements in antibiotic-resistant gene cassettes that can integrate into chromosomes or plasmids via site-specific recombination. Class 1 and class 2 integrons have been described in *A*. *baumannii* isolates that were associated with outbreaks of nosocomial infections [6]. Antibiotic resistance genes of *A*. *baumannii* that are located in integrons include *bla*_GES-14_, *bla*_IMP_, *bla*_VIM_, and *bla*_SIM_ [7]. Numerous studies have demonstrated that antibiotic resistance genes of *A*. *baumannii* that are located on plasmids include β-lactams (*bla*_GES-11_), carbapenems (*bla*_IMP_, *bla*_VIM_, *bla*_OXA-23_, *bla*_OXA-24_, *bla*_OXA-58_, and *bla*_NDM-1_), sulfonamide (*sul*2), and streptomycin (*str*AB) resistance genes [7, 8]. The primary features of the plasmids that have been circulating among *A*. *baumannii* strains were classified into homogeneous groups based on their genes controlling their replication systems (*rep* genes) by using PCR-based assays [9]. Among the 19 recently identified replicon groups, GR2, GR4, GR6, GR8, GR12, GR14, and GR16 were detected in the majority of *A*. *baumannii* clinical isolates. The GR6 plasmid was the most prevalent group detected in antibiotic-resistant *A*. *baumannii* (MDR-AB and CR-AB) [10]. The GR6 plasmid group can transfer antibiotic resistance genes, including *bla*_OXA-23_, *bla*_OXA-58_, and *aphA6*, among *Acinetobacter* spp. isolates [9, 11].

Antibiotic resistance genes with potential MGEs can be disseminated by horizontal gene transfer (HGT), which is occurs via three mechanisms, namely, transformation, conjugation, and transduction. Antimicrobial resistance genes are transferred in Gram-negative bacteria through conjugation, and conjugation-mediated transfer of antibiotic resistance genes has been reported in numerous studies [12–14]. However, few studies have reported the successful transfer of antibiotic resistance genes with integrons from clinical isolates of *Acinetobacter* spp. to environmental isolates through conjugation. The present study aimed to characterize the mechanisms underlying conjugation-mediated transfer of the antibiotic resistance genes from XDR-AB and NDM-AB to environmental isolates of *Acinetobacter* spp. by conducting *in vitro* conjugation experiments.

## Materials and methods

### Bacteria

Donor strains were extensively drug-resistant *A*. *baumannii* (XDR-AB) and New Delhi Metallo-beta-lactamase-1-producing *A*. *baumannii* (NDM-AB) isolated from four hospitals in Thailand based on a previously published study [15]. All isolates were collected by technical staff and microbiologists following routine procedures in the clinical microbiology laboratory of the four hospitals. A total of 14 isolates were selected as donors in the present study (S1 Table). The protocol was approved by Naresaun University Institutional Biosafety Committee (No. NUIBC GM 58-11-68).

### Isolation and identification of Acinetobacter species from environment

*Acinetobacter* spp. were isolated from water samples collected from Phitsanulok province in Thailand (S2 Table). The identification of the genus *Acinetobacter* was performed via biochemical testing. Molecular identification of the bacterial isolates was confirmed by DNA sequencing of the 16S rRNA or *rpo*B genes. Primer pairs for amplification of 16S rRNA and *rpo*B genes are listed in S3 Table [16, 17].

### Determination of antibiotic susceptibility

Kirby-Bauer diffusion method was used to determine the antibiotic susceptibility patterns of *A*. *baumannii* clinical isolates and environmental isolates of *Acinetobacter* spp. The following antibiotics were tested in the present study: amikacin (30 μg), cefepime (30 μg), cefotaxime (30 μg), cefoperazone/sulbactam (75 and 30 μg), ceftazidime (30 μg), ceftriaxone (30 μg), ciprofloxacin (5 μg), gentamicin (10 μg), imipenem (10 μg), meropenem (10 μg), piperacillin/tazobactam (100 and 10 μg), tetracycline (30 μg), tigecycline (15 μg), and trimethoprim/sulfamethoxazole (1.25 and 23.75 μg). Results of antibiotic susceptibility testing were interpreted according to the Clinical Laboratory Standard Institute **(**CLSI**)** [18]. The minimum inhibitory concentrations (MICs) of ticarcillin, kanamycin, tetracycline, and sodium azide were determined by conducting broth dilution tests [18].

### Replicon typing and detection of antibiotic resistance genes and integrons

Multiplex PCR and monoplex PCR assays were performed for the detection of antibiotic resistance genes using cell lysates, genomic DNA, or plasmids. Plasmids were extracted using PureDireX Plasmid miniPREP Kit (Bio-helix, Keelung, Taiwan). All isolates were tested for the presence of genes encoding class A, B, and D beta-lactamases, aminoglycoside resistance and tetracycline resistance, namely, *bla*_PER-1_, *bla*_OXA-23_, *bla*_OXA-24_, *bla*_OXA-58_, *bla*_NDM-1_, *aphA6*, *tet* (A) and *tet* (B) genes as previously described [19–23]. Identification of class 1, 2, and 3 integrons (*int1*, *int2*, *int3*) was performed following a previously described method [24–26]. Plasmid groups of *A*. *baumannii* were investigated by PCR-based replicon typing as described by Bertini *et al*. [9].

### Induction of azide-resistant *Acinetobacter* spp

*Acinetobacter* spp. environmental isolates that were found to be susceptible to all antibiotics tested were selected for induction of azide-resistant strains. The minimum inhibitory concentration (MIC) of sodium azide was determined. Then, spontaneous mutation to sodium azide was performed with continuous exposure *Acinetobacter* spp. to sodium azide as described by Randall *et al*. [27]. *Acinetobacter* spp. isolates with MIC values greater than or equal to 300 μg/ml were used as recipients.

### Conjugation assays

**Fig 1:**
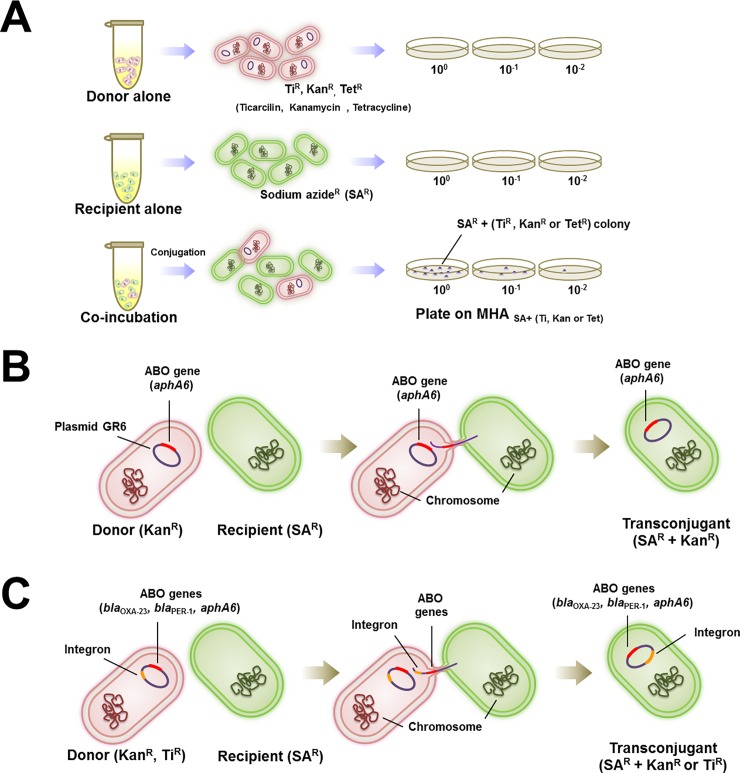
Conjugation experiments. (A) Experimental procedure for *in vitro* conjugation. (B) Horizontal gene transfer of plasmid group 6 (GR6) harboring the antibiotic resistance (ABO) genes between donor and recipient strains. (C) Horizontal gene transfer of untypeable GR carried integron and antibiotic resistance genes between donor and recipient strains.

Conjugation assays were performed to investigate the transfer of antibiotic resistance genes from *A*. *baumannii* clinical isolates to the environmental isolates (Fig 1A). A total of ten XDR-AB and four NDM-AB isolates were used as the donors. Overnight cultures of the donor and recipient cells were adjusted in 0.85% NaCl until a density corresponding to a McFarland value of 0.5 using a densitometer (SiaBiosan, Riga, Latvia). Afterwards, the donor and recipient cells were mixed at a ratio of 1:3 in Luria-Bertini (LB) broth and incubated for 4 h at 37°C. Transconjugants were recovered on MHA plates containing the following components: 300 μg/ml sodium azide; 50 μg/ml ticarcillin or 300 μg/ml sodium azide; 20 μg/ml tetracycline or 300 μg/ml sodium azide; and 20 μg/ml kanamycin. For the controls, the donors and recipients were each inoculated in LB broth and incubated for 4 h at 37°C, and the number of recipient cells (cfu) was calculated. The colonies that grew on the selective media were collected for the detection of antibiotic resistance genes, integrons, and resistance plasmid groups. Antibiotic susceptibility patterns and the MICs of ticarcillin, kanamycin, and tetracycline were determined to confirm the transfer of antibiotic resistance within all the transconjugants. Conjugation frequencies (CF) were calculated as follows:

$$CF=\underline{Number\;of\;transconjugants\;\left(cfu\right)\times dilution\;factor}$$

Numberofrecipients(cfu)

### S1-nuclease pulsed-field gel electrophoresis (S1-PFGE) and characterization of plasmids obtained from successful transconjugants

Bacterial cells were cultured in LB broth with shaking (150 rpm) at 37°C until the optical density reading (600 nm) of 0.8 was reached. Cells were harvested and suspended in Tris-EDTA (TE) buffer **(**100 mM Tris, 150 mM EDTA, pH 8.0**)**. The cell suspension was then mixed with 2% low melting point agarose and transferred into the plug molds. Plugs were lysed using TE buffer supplemented with 1 mg/ml proteinase K and 1% sodium dodecyl sulfate at 55°C in a shaking water bath for 4 h. Afterwards, plugs were washed thrice with 5 ml of washing buffer **(**1 mM Tris, 1 mM EDTA, pH 8.0**)** at 55°C. The plugs were treated with S1 nuclease (8 units/plug) **(**Thermo Fisher Scientific, Waltham, USA**)** at 25°C for 30 min. PFGE was performed using a CHEF Mapper XA system as previously described [28]. The gels were stained with ethidium bromide (5 μg/ml) for 20 min, and the band patterns were observed under a UV transilluminator. Linearized plasmid sizes were determined with Lambda PFG Ladder **(**New England Biolabs, Frankfurt, Germany**)**. Afterwards, the genomic and plasmid DNA of donor and transconjugant strains were cut and purified using GeneJET PCR Purification Kit (Thermo Fisher Scientific, illkirch, France). Detection of antibiotic resistance genes, integrase genes, and plasmid replicon types in purified DNA of transconjugants were performed using monoplex PCR and multiplex PCR methods.

## Results

### Isolation, identification, and characterization of *Acinetobacter* spp. isolated from environmental samples

A total of 19 *Acinetobacter* spp. were isolated from water samples. Species identification was performed by DNA sequencing of the 16S rRNA and *rpo*B genes. Nine (47.4%), eight (42.1%), and two isolates (10.5%) were assigned to the genus *Acinetobacter*, *A*. *baumannii*, *A*. *soli*, and *A*. *nosocomialis* (S2 Table). Out of 19 *Acinetobacter* spp. isolates, four (21.1%) were found to be susceptible to all the tested antibiotics. The four isolates did not acquire antibiotic resistance genes and integrons and were then selected for induction of azide resistance (S2 Table).

### Replicon typing and analysis of antibiotic susceptibility patterns, antibiotic resistance genes, and integrons in donor strains

XDR-AB donors were found to be resistant to all tested antibiotics, except for tigecycline. NDM-AB donors were resistant to β-lactam antibiotics, such as imipenem and meropenem. The *tet*(A), *bla*_OXA-24_, and *bla*_OXA-58_ genes were not detected in all 14 isolates. The *bla*_OXA-23_ gene was detected in all isolates, while the *bla*_NDM-1_, *tet*(B), *aphA6*, *bla*_PER-1_, and the integrase (*int1*) genes were detected in four, 12, four, four, and five isolates, respectively. Plasmid groups GR2 and GR6 were detected in the donor strains (S1 Table).

### Induction of azide resistance in *Acinetobacter* spp

Four *Acinetobacter* spp. isolates (NU005-1, NU013, NU015, and NI003) that were susceptible to all tested antibiotics were selected for induction of sodium azide resistance. Only two isolates (NU013R and NU015R) were successfully induced to sodium azide resistance with MIC values greater than 300 μg/ml and were subsequently used as recipients in this study. None of 19 plasmid groups was detected in the recipient strain NU013R. However, the plasmid group GR8 was detected in the recipient strain NU015R (Table 1).

**Table 1:** Conjugation frequencies (CFs), antibiotic susceptibility patterns, antibiotic resistance genes, integrase genes, and replicon typing of donors, recipients, and transconjugants.

**Isolates**^**a**^	**Selective antibiotics (CFs)**	**Drug susceptibility patterns**^**b**^	**PCR-based plasmid typing** **(GR)**^**c**^	**S1-PFGE based plasmid typing**^**d**^	**Transferred plasmids** **(GR**^**c**^ **or Size**^**d**^**)**	**Associated antibiotic resistance genes detected in cell lysate**
AB364 (Donor)	-	AK/CIP/SXT/CTX/CAZ/CSL/IPM/MEM/TZP/CRO/FEP/Ka/Ti	GR2	380 kb, 240 kb	-	*int1*, *bla*_OXA-23_, *bla*_NDM-1_, *aphA6*, *bla*_PER-1_, *tet***(**B**)**
AB140 (Donor)	-	AK/CIP/SXT/CTX/CAZ/CSL/IPM/MEM/TZP/TE/CRO/FEP/CN/Ka/Ti	GR2/GR6	Negative	-	*bla*_OXA-23_, *aphA6*, *tet***(**B**)**
AB352 (Donor)	-	AK/CIP/SXT/CTX/CAZ/IPM/MEM/TZP/CRO/FEP/Ka/Ti	GR2	380 kb, 220 kb	-	*int1*, *bla*_OXA-23_, *bla*_NDM-1_, *aphA6*, *bla*_PER-1_
AB405 (Donor)	-	AK/CIP/SXT/CTX/CAZ/CSL/IPM/MEM/TZP/TE/CRO/FEP/CN/Ka/Ti	GR2	220 kb	-	*int1*, *bla*_OXA-23_, *bla*_NDM-1_, *aphA6*, *bla*_PER-1_
**Isolates**^**a**^	**Selective antibiotics (CFs)**	**Drug susceptibility patterns**^**b**^	**PCR-based plasmid typing** **(GR)**^**c**^	**S1-PFGE based plasmid typing**^**d**^	**Transferred plasmids** **(GR**^**c**^ **or Size**^**d**^**)**	**Associated antibiotic resistance genes detected in plasmids**
NU013R (Recipient)	-	No resistance	Negative	Negative	-	-
NU013R-364 (TC09)	Ti (2.0 x 10^−5^)	AK/CTX/CAZ/CRO/FEP/CN/IPM/MEM/SXT/TZP/Ka/Ti	Negative	240 kb	240 kb **(**untypeable GR**)**	*int1*, *bla*_OXA-23_, *bla*_PER-1_
NU013R-364 (TC76)	Ka (4.5 x 10^−5^)	AK/CTX/CAZ/CRO/FEP/CN/IPM/MEM/SXT/TZP/Ka/Ti	Negative	240 kb	240 kb **(**untypeable GR**)**	*int1*, *bla*_OXA-23_, *bla*_PER-1_
NU013R-140 (TC12)	Ti (1.0 x 10^−4^)	AK/CTX/CAZ/CRO/FEP/TZP/Ka/Ti	GR6	Negative	GR6^e^	*aphA6*^f^
NU013R-140 (TC21)	Ka (8.5 x 10^−5^)	AK/CTX/CAZ/CRO/FEP/Ka/Ti	GR6	Negative	GR6^e^	*aphA6*^f^
NU013R-352 (TC31)	Ti (4.1 x 10^−7^)	AK/CTX/CAZ/CRO/FEP/CN/SXT/Ka/Ti	Negative	220 kb	220 kb **(**untypeable GR**)**	*int1*, *aphA6*, *bla*_PER-1_
NU013R-352 (TC41)	Ka (5.7 x 10^−7^)	AK/CTX/CAZ/CRO/FEP/CN/SXT/Ka/Ti	Negative	220 kb	220 kb **(**untypeable GR**)**	*int1*, *aphA6*, *bla*_PER-1_
NU013R-405 (TC51)	Ti (3.7 x 10^−5^)	AK/CTX/CAZ/CRO/FEP/CN/SXT/Ka/Ti	Negative	220 kb	220 kb **(**untypeable GR**)**	*int1*, *aphA6*, *bla*_PER-1_
NU013R-405 (TC61)	Ka (1.3 x 10^−5^)	AK/CTX/CAZ/CRO/FEP/CN/SXT/Ka/Ti	Negative	220 kb	220 kb **(**untypeable GR**)**	*int1*, *aphA6*, *bla*_PER-1_
NU015R (Recipient)	-	No resistance	GR8	485 kb	-	-
NU015R-364 (TC02)	Ti (3.7 x 10^−5^)	AK/CTX/CAZ/CRO/FEP/CN/IPM/MEM/SXT/TZP/Ka/Ti	GR8	485 kb, 240 kb	240 kb **(**untypeable GR**)**	*int1*, *bla*_OXA-23_, *bla*_PER-1_
NU015R-364 (TC71)	Ka (9.6 x 10^−5^)	AK/CTX/CAZ/CRO/FEP/CN/IPM/MEM/SXT/TZP/Ka/Ti	GR8	485 kb, 240 kb	240 kb **(**untypeable GR**)**	*int1*, *bla*_OXA-23_, *bla*_PER-1_
NU015R-140 (TC16)	Ti (8.6 x 10^−5^)	AK/CTX/CAZ/CRO/Ka/Ti	GR8/GR6	485 kb	GR6^e^	*aphA6*^f^
NU015R-140 (TC26)	Ka (6.3 x 10^−5^)	AK/CTX/CAZ/CRO/FEP/Ka/Ti	GR8/GR6	485 kb	GR6^e^	*aphA6*^f^
NU015R-352 (TC36)	Ti (9.5 x 10^−6^)	AK/CTX/CAZ/CRO/FEP/CN/SXT/Ka/Ti	GR8	485 kb, 220 kb	220 kb **(**untypeable GR**)**	*int1*, *aphA6*, *bla*_PER-1_
NU015R-352 (TC46)	Ka (2.3 x 10^−5^)	AK/CTX/CAZ/CRO/FEP/CN/SXT/Ka/Ti	GR8	485 kb, 220 kb	220 kb **(**untypeable GR**)**	*int1*, *aphA6*, *bla*_PER-1_
NU015R-405 (TC56)	Ti (5.2 x 10^−5^)	AK/CTX/CAZ/CRO/FEP/CN/SXT/Ka/Ti	GR8	485 kb, 220 kb	220 kb **(**untypeable GR**)**	*int1*, *aphA6*, *bla*_PER-1_
NU015R-405 (TC66)	Ka (4.7 x 10^−5^)	AK/CTX/CAZ/CRO/FEP/CN/SXT/Ka/Ti	GR8	485 kb, 220 kb	220 kb **(**untypeable GR**)**	*int1*, *aphA6*, *bla*_PER-1_

^a^TC: transconjugants

^b^AK: amikacin, FEP: cefepime, CTX: cefotaxime, CSL: cefoperazone/ sulbactam, CAZ: ceftazidime, CRO: ceftriaxone, CIP: ciprofloxacin, CN: gentamicin, IPM: imipenem, MEM: meropenem, TZP: piperacillin/tazobactam, TE: tetracycline, SXT: trimethoprim/sulfamethoxazole, Ka: kanamycin, Ti: ticarcillin

^c^Plasmid group determined by PCR-based plasmid typing

^d^Plasmid size analyzed by S1-PFGE-based method

^e^This plasmid GR was detected by the PCR-based method but could not detected by the S1-PFGE method

^f^ This gene was amplified in plasmids extracted using PureDireX Plasmid miniPREP Kit **(**Bio-helix, Keelung, Taiwan**)**.

### Conjugation assays

Successful transconjugants were generated from four (AB364, AB140, AB352, and AB405) out of the 14 donor strains. The conjugation frequencies (CFs) of the strains are listed in Table 1. The transconjugant colonies were detected on plates containing 50 μg/ml ticarcillin and sodium azide and occurred at frequencies ranging from 1.0 × 10^−4^ to 4.1 × 10^−7^. The CFs of kanamycin-resistant strains detected on plates containing 20 μg/ml kanamycin and sodium azide ranged between 1.3 ×10^−5^ and 5.7 ×10^−7^. No transconjugants were detected on MHA plates containing 20 μg/ml tetracycline.

### Antibiotic resistance genes, antibiotic susceptibility patterns, minimum inhibitory concentrations (MICs) of transconjugants

Antibiotic resistance genes were detected in the selected transconjugants. Results indicated that the donor strain AB364 can transfer both the *bla*_OXA-23_ and *bla*_PER-1_ genes to both recipients. The strains AB352 and AB405 can transfer both *aphA6* and *bla*_PER-1_ genes from the donor strains to both recipients **(**Table 1**)**. AB140 can only transfer the *aphA6* gene to both recipients **(**Table 1**).** Our current findings indicated that the *bla*_NDM-1_ and *tet*(B) genes cannot be transferred by conjugation **(**Table 1**)**. The integrase gene of class 1 integrons (*int1*) can be transferred from the donor strains AB364, AB352, and AB405 to all transconjugants. The PCR products corresponding to the *bla*_OXA-23_, *aphA6*, *bla*_PER-1,_ and *int1* genes from the transconjugants derived from the conjugation experiments are presented in S1 Fig. Only the GR6 plasmid group was transferred from the donor strain AB140 to its transconjugants (Table 1). The GR2 plasmid group was observed in all four donors and was determined to be a non-transferable plasmid. Results from disc diffusion testing reveled that all transconjugants were multidrug-resistant strains (Table 1). MIC against ticarcillin of transconjugants was greater than 256 μg/ml. MIC of kanamycin was 128 μg/ml, which is around six-fold higher the MIC of kanamycin on the recipient strains.

### Plasmids transferred from transconjugants and their associated antibiotic resistance genes

The results of S1-PFGE based plasmid typing are presented in Table 1. The AB364 strain harbored two different mega-plasmids with sizes of 380 kb and 240 kb. The AB352 strain harbored 380-kb and 220-kb plasmids. The AB405 strain carried only a 220-kb plasmid. No mega-plasmids were detected in AB140 using the S1-PFGE method (S2 Fig). Conjugation experiments demonstrated that the 240-kb plasmid was successfully transferred from AB364 into both recipient strains; however, the GRs of transferred plasmids were untypeable. Three genes (*int1*, *bla*_OXA-23_, and *bla*_PER-1_) were detected in the transferred plasmids. Moreover, plasmids from the AB352 and AB405 strains (220 kb) can be transferred into both recipients. PCR analysis showed that the *int1*, *aphA6*, and *bla*_PER-1_ genes associated with antibiotic resistance were present in the two 220 kb plasmids. In addition, the *aphA6* gene associated with the GR6 plasmid could be transferred from AB140 to both recipients.

## Discussion

Conjugation assay of antibiotic resistance genes can be performed with various recipients. Numerous studies have reported successful conjugations from *A*. *baumannii* to *E*. *coli* J53 [29, 30]. However, interspecies gene transfer mediated by conjugation confers genetic barriers [31]. In the present study, we isolated two sodium azide-resistant strains without antibiotic resistance, which were successfully used as recipients for conjugation experiments. Results of conjugation experiments demonstrated that resistance to ticarcillin and kanamycin could be transferred from four XDR-AB donors to two sodium azide-resistant *A*. *baumannii* isolates. The CF for ticarcillin resistance ranges between 1.0 × 10^−4^ and 4.1 × 10^−7^, which were found to be higher than those reported in a previous study by Zarrilli *et al*. and Krahn *et al*. (2 × 10^−5^ and 1.0 × 10^−6^) [32, 33]. The CFs for kanamycin resistance ranged from 1.3 × 10^−5^ to 5.7 × 10^−7^. In contrast to previous findings, in which *tet*(E) and *tet*(B) genes were transferred from MDR-AB to *E*. *coli* J53, no transconjugants were detected on MHA plates containing tetracycline in the present study [29]. Donors, recipients, and transconjugants were selected to investigate their genetic diversity by conducting repetitive element palindromic-PCR **(**rep-PCR**)**. The banding patterns of transconjugants were similar to both recipients, thereby indicating similar genotypes between the transconjugants and the recipients **(**S3 Fig**)**.

MGEs are attributed to the evolution and genetic variation of bacterial niches [34]. The transfer of antibiotic resistance elements harboring potential MGEs, such as plasmids, integrons, and conjugative transposons, is an important mechanism involved in the emergence of antibiotic-resistant bacteria. In the present study, two plasmid groups (GR2 and GR6) were identified among four donor strains (Table 1). Only the GR6 plasmid was successfully transferred by conjugation to both recipient strains. The GR6 plasmid belongs to a group of low-copy-number plasmids, considering that it cannot be detected by S1-PFGE-based plasmid typing but could be detected by PCR-based plasmid typing. Previous studies identified GR6 as the main replicon plasmid group in *A*. *baumannii* that harbored β-lactamase genes (*bla*_OXA-23_, *bla*_OXA-40/24_, and *bla*_OXA-58_) [9, 10]. In the present study, plasmids were extracted from transconjugants and used as templates for the detection of plasmid groups and antibiotic resistance genes. We detected the successful transfer of the GR6 plasmid with kanamycin resistance gene (*aphA6*) to both recipients (Table 1 and Fig 1B). Likewise, Hamidian *et al*. reported that the *aphA6* gene, which was detected in conjugative plasmids, belongs to the Aci6 *Acinetobacter* plasmid family [11], which is associated with the *tra* locus and is likely to be involved in plasmid mobilization and HGT among *A*. *baumannii* [10].

Another phenomenon that we observed in conjugation study is that the integron associated transfer with antibiotic resistance genes. Many studies have reported the high prevalence of class 1 integrons among *A*. *baumannii* [24]. In the present study, the integrase gene (*int1*) was detected in three donor strains used for conjugation, and the presence of *int1* gene was additionally confirmed in 12 transconjugant strains (Table 1). Class 1 integrons can insert themselves into conjugative plasmids or transposons. Results of conjugation assay suggested that integrons could be co-transferred with antibiotic resistance genes from plasmids to recipient strains (Fig 1C). Our findings revealed that the donor AB364 strain can co-transfer the *bla*_OXA-23_ and *bla*_PER-1_ genes with the *int1* gene to both recipients. Donor AB364 harbored two different mega-sized plasmids (380 kb and 240 kb); however, only the 240-kb plasmid (untypeable group) can be transferred to both recipients based on S1-PFGE-based plasmid typing. The *bla*_OXA-23_, *bla*_PER-1_, and *int1* genes were detected by PCR using the 240-kb plasmid as template, which indicated the transfer of two antibiotic resistance genes in association with integron through this plasmid. Consistent with our current findings, Karah *et al*. reported that *bla*_GES_ genes were located in integron cassettes, although this conjugative transferability remains to be characterized [35]. In contrast to the donor AB364 strain, only the 220-kb (untypeable group) plasmids from donor AB352 and AB405 can be transferred to both recipients (Table 1). The transfer of *aphA6* and *bla*_PER-1_ genes in association with *int1* can be detected in this mega-plasmid.

None of the four *A*. *baumannii* donor strains tested were able to transfer the tetracycline resistance gene *tet*(B) to recipient strains. Numerous studies have demonstrated that the tetracycline resistance genes *tet*(A) and *tet*(O) could be transferred among bacteria primarily though transduction [36,37]. Furthermore, successful transfer of the *bla*_NDM-1_ gene was not detected in the present study, which could be explained by the *bla*_NDM-1_ gene of the *bla*_NDM-1_-producing *A*. *baumannii* that was located on the chromosome [33]. Furthermore, Chatterjee *et al*. demonstrated that the *bla*_NDM-1_ gene in *A*. *baumannii* was successfully transferred to both *E*. *coli* JM109 and *A*. *baumannii* ATCC19606 through outer membrane vesicles (OMVs), with transformation frequencies ranging from 10^−5^ to 10^−6^ [38].

In conclusion, we generated two environmental isolates, namely, NU013R and NU015R, as recipient strains. Results revealed that the *bla*_OXA-23_, *bla*_PER-1_, and *aphA6* genes could be transferred between *A*. *baumannii* clinical isolates and *A*. *baumannii* environmental isolates via the plasmid group GR6 or class 1 integrons through *in vitro* conjugation. Consequently, the mechanism underlying gene transfer is a potential factor responsible for the rapid spread of antibiotic-resistant bacteria worldwide.

## Supporting information

S1 Table Donor strains used in this study. (DOCX)

S2 Table Environmental isolated strains used in this study. (DOCX)

S3 Table List of primers used in this study. (DOCX)

S1 Fig PCR product to detect antibiotic resistance genes in donor, recipient and transconjugant strains. (A) Lane M: 100 bp ladder, Lane N: Negative control, Lane 1: PCR product of positive for *bla*_OXA-51_ and *bla*_OXA-23_ genes from donor, Lane 2–3: PCR product of *bla*_OXA-51_ from recipient NU013R and NU015R, respectively, Lane 4–5: PCR product of *bla*_OXA-51_ and *bla*_OXA-23_ genes from tranconjugants. (B) Lane M: 100 bp ladder, Lane N: Negative control, Lane 1: PCR product of positive for *bla*_OXA-51_ and *aphA6* genes from donors, Lane 2–3: PCR product of *bla*_OXA-51_ from recipient NU013R and NU015R, respectively, Lane 4–5: PCR product of *bla*_OXA-51_ and *aphA6* genes from tranconjugants. (C) Lane M: 100 bp ladder, Lane N: Negative control, Lane 1: PCR product of positive for *bla*_OXA-51_ and *bla*_PER-1_ genes from donors, Lane 2–3: PCR product of *bla*_OXA-51_ from recipient NU013R and NU015R, respectively, Lane 4–5: PCR product of *bla*_OXA-51_ and *bla*_PER-1_ genes from tranconjugants. (D) Lane M: 100 bp ladder, Lane N: Negative control, Lane 1: PCR product of positive for *bla*_OXA-51_ and *int1* genes from donors, Lane 2–3: PCR product of *bla*_OXA-51_ from recipient NU013R and NU015R, respectively, Lane 4–5: PCR product of *bla*_OXA-51_ and *int1* genes from tranconjugants.
(TIF)

S2 Fig Results of transferred plasmids obtained from successful transconjugants using S1-PFGE. Lane M: Lambda PFG Ladder, as the marker, Lane 1: S1-PFGE profile of NU013R as a recipient, Lane 2–5: S1-PFGE profiles of four transconjugants isolates, NU013R-364, NU013R-140, NU013R-352, NU013R-405, respectively. Lane 6: S1-PFGE profile of NU015R as a recipient, Lane 7–10: S1-PFGE profiles of four transconjugants isolates, NU015R-364, NU015R-140, NU015R-352, NU015R-405, respectively.
(TIF)

S3 Fig Repetitive element palindromic-PCR (Rep-PCR) analysis of donor, recipient and transconjugant strains. Lane M: 1 kb ladder, Lane 1–2: *A*. *baumannii* donor, Lane 3: *Acinetobacter* spp. recipient, NU013R, Lane 4: *Acinetobacter* spp. recipient, NU015R, Lane 5,7: *Acinetobacter* spp. NU013R transconjugant strains, Lane 6,8: *Acinetobacter* spp. NU015R transconjugant strains.
(TIF)
