# Supplementary material for: Acquisition and transfer of antibiotic resistance genes in association with conjugative plasmid or class 1 integrons of Acinetobacter baumannii
Source: PLoS One. 2018 Dec 6;13(12):e0208468. doi: 10.1371/journal.pone.0208468 (PMC6283642; doi:10.1371/journal.pone.0208468)
Supplement: S1 Table — (DOCX) [file pone.0208468.s001.docx]

**S1 Table. Donor strains used in this study.**

| **Strains** | **Source of isolates** | **Rep-PCR** | **Plasmid groups** | **Type of integrase** | **Antibiotic resistance genes** | | | | |
| --- | --- | --- | --- | --- | --- | --- | --- | --- | --- |
|  |  |  |  |  | ***bla*_OXA-23_** | ***bla*_NDM-1_** | ***tet***(**B**) | ***aphA6*** | ***bla*_PER-1_** |
| AB135 | pus | A | GR2/GR6 | **-** | + | **-** | + | **-** | **-** |
| AB136 | urine | B | GR2 | *int1* | + | **-** | + | **-** | **+** |
| AB140 | sputum | A | GR2/GR6 | **-** | + | **-** | + | + | **-** |
| AB229 | urine | A | GR2/GR6 | **-** | + | **-** | + | **-** | **-** |
| AB095 | sputum | A | GR2 | **-** | + | **-** | + | **-** | **-** |
| AB180 | sputum | A | GR2 | **-** | + | **-** | + | **-** | **-** |
| AB183 | sputum | A | GR2 | **-** | + | **-** | + | **-** | **-** |
| AB241 | sputum | A | GR2 | **-** | + | **-** | + | **-** | **-** |
| AB246 | sputum | A | GR2 | **-** | + | **-** | + | **-** | **-** |
| AB269 | sputum | A | GR2 | **-** | + | **-** | + | **-** | **-** |
| AB120 | sputum | C | Untypeable | *int1* | + | + | + | **-** | **-** |
| AB352 | sputum | D | GR2 | *int1* | + | + | **-** | + | + |
| AB364 | ascitic swab | D | GR2 | *int1* | + | + | + | + | + |
| AB405 | sputum | C | GR2 | *int1* | + | + | **-** | + | + |
