# Supplementary material for: Acquisition and transfer of antibiotic resistance genes in association with conjugative plasmid or class 1 integrons of Acinetobacter baumannii
Source: PLoS One. 2018 Dec 6;13(12):e0208468. doi: 10.1371/journal.pone.0208468 (PMC6283642; doi:10.1371/journal.pone.0208468)
Supplement: S2 Table — (DOCX) [file pone.0208468.s002.docx]

**S2 Table. Environmental isolated strains used in this study.**

| **Strains** | **Species** | **Antibiotic susceptibility patterns^a^** | **Susceptibility categories^b^** | | | **Antibiotic resistance genes** | | | | |
| --- | --- | --- | --- | --- | --- | --- | --- | --- | --- | --- |
|  |  |  | **Ticarcillin 50 µg/ml** | **Kanamycin 20 µg/ml** | **Tetracycline 20 µg/ml** | ***bla*_OXA-23_** | ***bla*_NDM-1_** | ***tet*(B)** | ***aphA6*** | ***bla*_PER-1_^c^** |
| NU001-1 | *A. baumannii* | No resistant | R | S | S | - | **-** | - | **-** | ND |
| NU002-1 | *A. baumannii* | CTX,CAZ, CRO,FEP,CIP,  CN,IPM,MEM,SXT,SCF,PIP | R | R | R | - | **-** | - | **-** | ND |
| NU003-1 | *A. soli* | No resistant | R | R | R | - | **-** | - | - | ND |
| NU003-2 | *A. soli* | No resistant | R | R | R | - | **-** | - | **-** | ND |
| NU003-3 | *A. soli* | No resistant | R | S | S | - | **-** | - | **-** | ND |
| NU005-1 | *A. nosocomialis* | No resistant | S | S | S | - | **-** | - | **-** | - |
| NU005-2 | *A. soli* | No resistant | R | S | S | - | **-** | - | **-** | ND |
| NU006-4 | *A. soli* | No resistant | R | S | S | - | **-** | - | **-** | ND |
| NU006-5 | *A. soli* | SXT | R | S | S | - | **-** | - | **-** | ND |
| NU006-8 | *A. soli* | No resistant | R | S | S | - | **-** | - | **-** | ND |
| NU006-9 | *A. soli* | No resistant | R | S | S | - | - | - | **-** | ND |
| NU009 | *A. baumannii* | AK,CTX,CAZ,CRO,FEP,  CIP,CN,MEM, SXT,PIP | R | R | R | + | - | **+** | - | ND |
| NU010-1 | *A. nosocomialis* | No resistant | R | S | S | + | - | + | - | ND |
| NU010-2 | *A. baumannii* | AK,CTX,CAZ,CRO,FEP,  CIP,CN,IPM, MEM, PIP | R | R | R | + | - | **+** | - | ND |
| NU010-3 | *A. baumannii* | CTX,CAZ,CRO,FEP,CIP,CN,  IPM,MEM, SXT,TE,PIP | R | S | R | - | - | **-** | - | ND |
| NU010-4 | *A. baumannii* | AK,CTX,CAZ,CRO,FEP,CIP,  CN,IPM,MEM,SXT,TE,PIP | R | R | R | + | - | **+** | - | ND |
| NU013 | *A. baumannii* | No resistant | S | S | S | - | - | **-** | - | - |
| NU015 | *A. baumannii* | No resistant | S | S | S | - | - | **-** | - | - |
| NI003 | *A. baumannii* | No resistant | S | S | S | - | - | **-** | - | - |

**^a^**AK: amikacin, FEP: cefepime, SCF: cefoperazone/sulbactam, CTX: cefotaxime, CAZ: ceftazidime, CRO: ceftriaxone, CIP: ciprofloxacin, CN: gentamicin, IPM: imipenem, MEM: meropenem, PIP: piperacillin/tazobactam, TE: tetracycline, SXT: trimethoprim/sulfamethoxazole.

**^b^**R: Resistant, S: Susceptible.

^c^ND: Not determine.
