# Supplementary material for: Acquisition and transfer of antibiotic resistance genes in association with conjugative plasmid or class 1 integrons of Acinetobacter baumannii
Source: PLoS One. 2018 Dec 6;13(12):e0208468. doi: 10.1371/journal.pone.0208468 (PMC6283642; doi:10.1371/journal.pone.0208468)
Supplement: S3 Table — (DOCX) [file pone.0208468.s003.docx]

**S3 Table. List of primers used in this study.**

| **Target genes** | **Primer name** | **Sequence (5’-3’)** | **Amplicon size (bp)** | **Annealing temp.**  **(^o^C)** | **References** |
| --- | --- | --- | --- | --- | --- |
| *16S rRNA* | 16s rRNA F | AGAGTTTGATCCTGGCTCAG | 1,500 | 58 | [16] |
|  | 16s rRNA R | ACGGCTACCTTGTTACGACTT |  |  |  |
| *rpo*B | rpoB F | CCTTCATGACCTGGAAYGGNTA | 940 | 59 | [17] |
|  | rpoB R | TCCAGGATCTGNCCNACRTTCAT |  |  |  |
| *bla*_OXA-51_ | blaOXA-51 F | TAATGCTTTGATCGGCCTTG | 353 | 52 | [19] |
|  | blaOXA-51 R | TGGATTGCACTTCATCTTGG |  |  |  |
| *bla*_OXA-23_ | blaOXA-23 F | GATCGGATTGGAGAACCAGA | 501 | 52 | [19] |
|  | blaOXA-23 R | ATTTCTGACCGCATTTCCAT |  |  |  |
| *bla*_OXA-24_ | blaOXA-24 F | GGTTAGTTGGCCCCCTTAAA | 246 | 52 | [19] |
|  | blaOXA-24 R | AGTTGAGCGAAAAGGGGATT |  |  |  |
| *bla*_OXA-58_ | blaOXA-58 F | AAGTATTGGGGCTTGTGCTG | 599 | 52 | [19] |
|  | blaOXA-58 R | CCCCTCTGCGCTCTACATAC |  |  |  |
| *bla*_NDM-1_ | blaNDM-1 F | GGTTTGGCGATCTGGTTTTC | 621 | 52 | [21] |
|  | blaNDM-1 R | CGGAATGGCTCATCACGATC |  |  |  |
| *bla*_PER-1_ | blaPER-1 F | ATGAATGTCATTATAAAAGC | **900** | 52 | [20] |
|  | blaPER-1 R | AATTTGGGCTTAGGGCAAGAAA |  |  |  |
| *tet***(**A**)** | tetA F | GCGCGATCTGGTTCACTCG | 164 | 61 | [22] |
|  | tetA R | AGTCGACAGYRGCGCCGGC |  |  |  |
| *tet***(**B**)** | tetB F | TACGTGAATTTATTGCTTCGG | 206 | 61 | [22] |
|  | tetB R | ATACAGCATCCAAAGCGCAC |  |  |  |
| *aphA6* | aphA6 F | ATGGAATTGCCCAATATTATTC | 736 | 55 | [23] |
|  | aphA6 R | TCAATTCAATTCATCAAGTTTTA |  |  |  |
| *int 1* | int1 F | CAGTGGACATAAGCCTGTTC | 160 | 52 | [25] |
|  | int1 R | CCCGAGGCATAGACTGTA |  |  |  |
| *int 2* | int2 F | TTGCGAGTATCCATAACCTG | 288 | 52 | [25] |
|  | int2 R | TTACCTGCACTGGATTAAGC |  |  |  |
| *int 3* | int3 F | GCCTCCGGCAGCGACTTTCAG | 979 | 56 | [24] |
|  | int3 R | ACGGATCTGCCAAACCTGACT |  |  |  |
