# Supplementary figures and images for: Acquisition and transfer of antibiotic resistance genes in association with conjugative plasmid or class 1 integrons of Acinetobacter baumannii
Source: PLoS One. 2018 Dec 6;13(12):e0208468. doi: 10.1371/journal.pone.0208468 (PMC6283642; doi:10.1371/journal.pone.0208468)

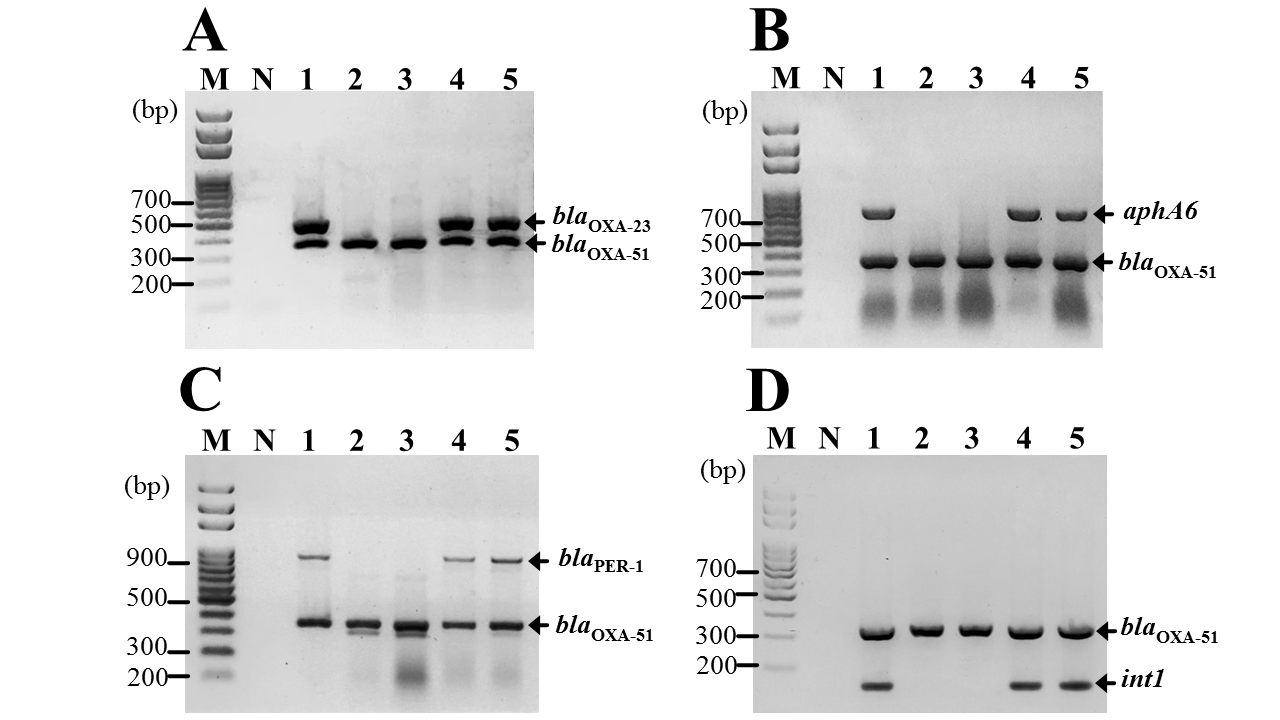

Supplement: S1 Fig — (A) Lane M: 100 bp ladder, Lane N: Negative control, Lane 1: PCR product of positive for blaOXA-51 and blaOXA-23 genes from donor, Lane 2–3: PCR product of blaOXA-51 from recipient NU013R and NU015R, respectively, Lane 4–5: PCR product of blaOXA-51 and blaOXA-23 genes from tranconjugants. (B) Lane M: 100 bp ladder, Lane N: Negative control, Lane 1: PCR product of positive for blaOXA-51 and aphA6 genes from donors, Lane 2–3: PCR product of blaOXA-51 from recipient NU013R and NU015R, respectively, Lane 4–5: PCR product of blaOXA-51 and aphA6 genes from tranconjugants. (C) Lane M: 100 bp ladder, Lane N: Negative control, Lane 1: PCR product of positive for blaOXA-51 and blaPER-1 genes from donors, Lane 2–3: PCR product of blaOXA-51 from recipient NU013R and NU015R, respectively, Lane 4–5: PCR product of blaOXA-51 and blaPER-1 genes from tranconjugants. (D) Lane M: 100 bp ladder, Lane N: Negative control, Lane 1: PCR product of positive for blaOXA-51 and int1 genes from donors, Lane 2–3: PCR product of blaOXA-51 from recipient NU013R and NU015R, respectively, Lane 4–5: PCR product of blaOXA-51 and int1 genes from tranconjugants. (TIF) [file pone.0208468.s004.tif]

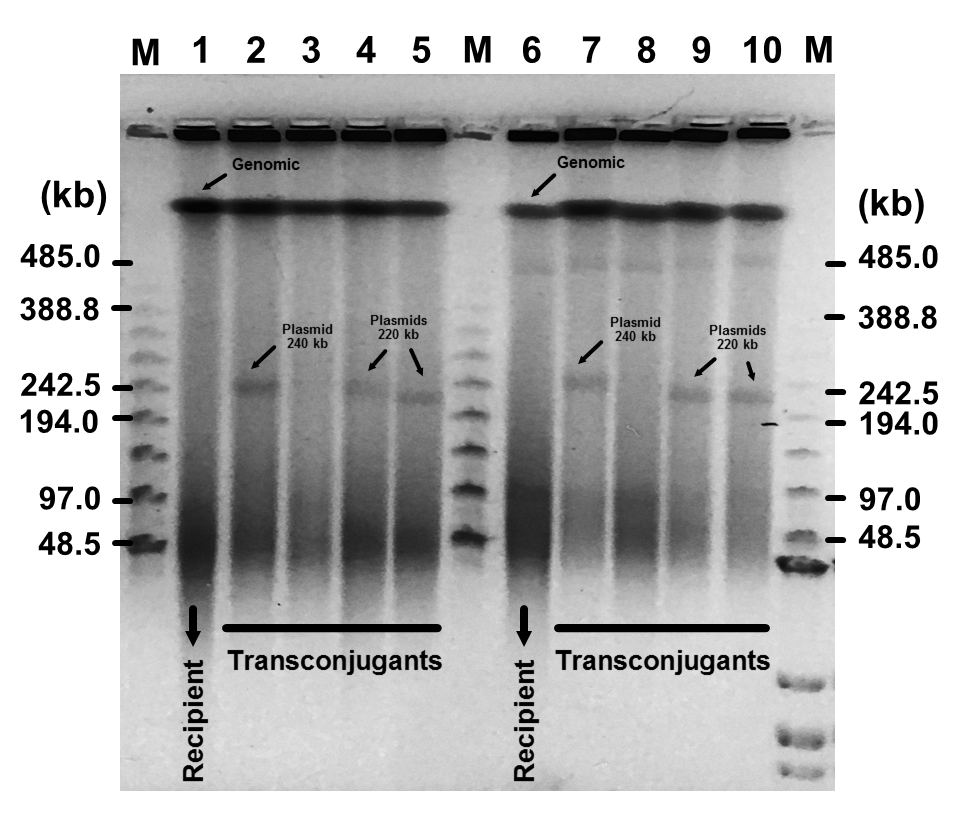

Supplement: S2 Fig — Lane M: Lambda PFG Ladder, as the marker, Lane 1: S1-PFGE profile of NU013R as a recipient, Lane 2–5: S1-PFGE profiles of four transconjugants isolates, NU013R-364, NU013R-140, NU013R-352, NU013R-405, respectively. Lane 6: S1-PFGE profile of NU015R as a recipient, Lane 7–10: S1-PFGE profiles of four transconjugants isolates, NU015R-364, NU015R-140, NU015R-352, NU015R-405, respectively. (TIF) [file pone.0208468.s005.tif]

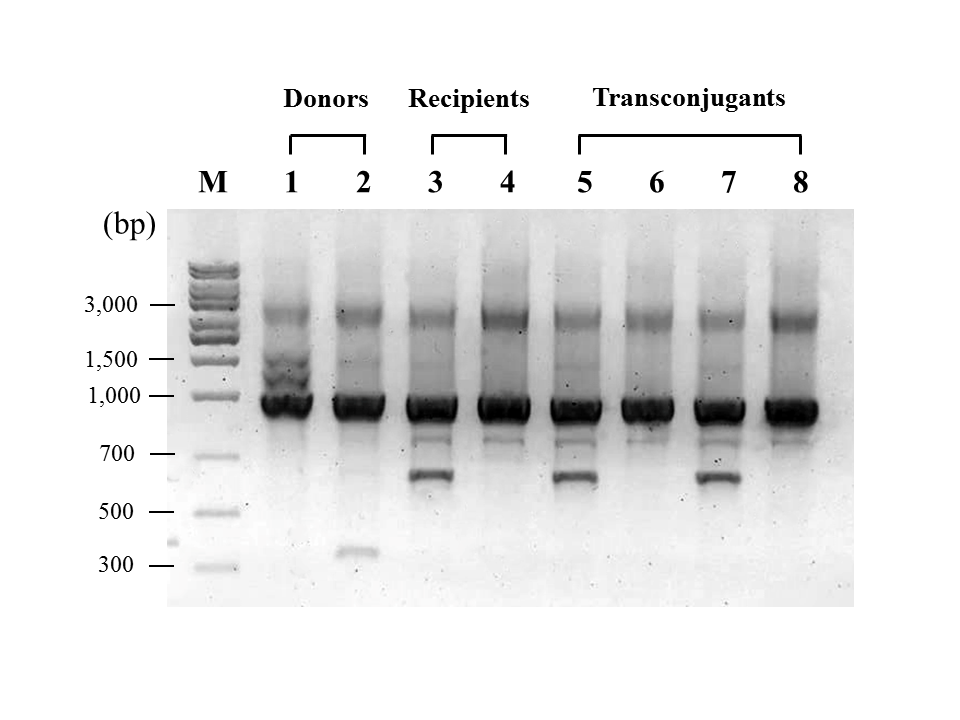

Supplement: S3 Fig — Lane M: 1 kb ladder, Lane 1–2: A. baumannii donor, Lane 3: Acinetobacter spp. recipient, NU013R, Lane 4: Acinetobacter spp. recipient, NU015R, Lane 5,7: Acinetobacter spp. NU013R transconjugant strains, Lane 6,8: Acinetobacter spp. NU015R transconjugant strains. (TIF) [file pone.0208468.s006.tif]
